# First Data on the Investigation of Gut Yeasts in Hermit Beetle (*Osmoderma barnabita* Motschulsky, 1845) Larvae in Lithuania

**DOI:** 10.3390/jof10070442

**Published:** 2024-06-22

**Authors:** Jurgita Švedienė, Vita Raudonienė, Goda Mizerienė, Jolanta Rimšaitė, Sigitas Algis Davenis, Povilas Ivinskis

**Affiliations:** 1 Laboratory of Biodeterioration Research, Nature Research Centre, 08412 Vilnius, Lithuania; vita.raudoniene@gamtc.lt; 2 Laboratory of Plant Pathology, Nature Research Centre, 08412 Vilnius, Lithuania; goda.mizeriene@gamtc.lt; 3 Laboratory of Entomology, Nature Research Centre, 08412 Vilnius, Lithuania; seni.medziai@gmail.com (J.R.); algis.davenis@gamtc.lt (S.A.D.); entlab@gmail.com (P.I.)

**Keywords:** yeasts, beetle, larvae, gut, diversity, identification

## Abstract

In this study, yeasts from the gut of *O. barnabita* larvae were isolated and molecularly identified. It is worth noting that this research provides the first analysis of the gut yeast community in *O. barnabita* larvae in Lithuania, which is a significant contribution to the field. Two hermit-like L3-praepupa instars were collected from a decaying oak log in Lithuania. The isolation, morphology, biochemistry, and physiology of the yeast isolates were characterized using standards commonly employed in yeast taxonomy studies. The isolates were identified by sequencing the large subunit (26S) rDNA (D1/D2 domain of the LSU). All gut compartments were colonized by the yeast. A total of 45 yeast strains were obtained from the gut of both *O. barnabita* larvae, with 23 strains originating from Larva 1, 16 strains from Larva 2, and 6 strains from the galleries. According to our identification results of the 45 yeast strains, most of the species were related to *Ascomycota*, with most of them belonging to the *Saccharomycetales* order. Yeasts of the genera *Candida*, *Debaryomyces*, *Meyerozyma*, *Priceomyces*, *Schwanniomyces*, *Spencermartinsiella*, *Trichomonascus*, and *Blastobotrys* were present in gut of *O. barnabita* larvae. Species of the *Trichosporonales* order represented the *Basidiomycota* phylum.

## 1. Introduction

Veteran deciduous trees, which have existed for a long time, are rare and highly specific habitats. In Europe, including Lithuania, they are home to a wide variety of animals, especially invertebrates, as well as fungi and lichens. It is worth noting that dead or decaying wood actually represents a significant pool of organic C, energy, and other nutrients [1]. The *Osmoderma* chafers are a type of saproxylic insect that can be found inhabiting hollows in deciduous trees, including oaks, ashes, limes, and beeches [2,3,4,5,6,7]. They belong to the family Cetoniidae (order Coleoptera). In several countries, *Osmoderma* species are classified as endangered [8,9,10,11,12,13,14,15]. Extensive research has been conducted on this genus of beetles in Europe, with a particular focus on their biology, life histories, natural enemies, and habitat preferences. However, this research has rarely been carried out in other regions.

The hermit beetle (*Osmoderma barnabita*) is a species commonly associated with primeval broad-leaved forests. It is known to serve as an umbrella species for many invertebrates found in such forests, particularly those with old hollow trees [16]. This beetle has experienced a significant decline across its distribution range, with reports of extinction in some countries due to habitat loss and intensive forest management. The current population trend of this species in Europe is decreasing [17,18]. It is worth noting that the species is classified as vulnerable in the Lithuanian Red Data Book [19], is also included in the Bern Convention (Annex II) and the EU Habitat Directive (Annexes II and IV), and is an indicator species of key forest habitats [20]. The mycobiome of the beetle presents an opportunity for advanced research in symbiosis due to its diverse nature, widespread availability, and replication of evolutionary origins [21].

Insects living in dead or decaying wood are often closely associated with microorganisms [22]. The gut of insects usually contains a highly diverse microflora, including various bacteria, yeasts, and protists. It has been suggested that microorganisms play an integral role in their hosts, including influencing the host metabolism, providing essential amino acids, vitamins, and nitrogen to the host, promoting the efficient digestion of nutrient-poor diets and recalcitrant foods, supporting defense and detoxification capabilities, and protecting hosts from potentially harmful microbes [23,24,25].

Yeasts are a unique group of fungi and inhabit all aerobic environments, from the Arctic and glaciers to the tropics or even the desert, and from arid to saline and sugar-rich habitats [26,27,28,29,30,31]. Many yeast species that are found in living or decaying plant parts are associated with insects [32]. More than 650 yeasts belonging to the phyla Ascomycota and Basidiomycota have been reported from the gut of beetles [33]. Yeasts from the genus *Alloascoidea* and ascomycete *Ophiostoma* play essential nutritional roles in facultative and obligate mutualisms with bark, ambrosia, and ship-timber beetles [23].

The current knowledge regarding the yeasts associated with gut systems and their importance to the hermit beetle’s larvae is limited, as the species is listed as endangered in many countries. Therefore, the main aim of this study was to isolate and identify the yeasts from the gut of *O. barnabita* larvae. It is worth noting that this research provides the first analysis of the gut yeast community of *Osmoderma barnabita* larvae in Lithuania, which is a significant contribution to the field. Improving our understanding of *O. barnabita*–fungal (yeast) symbiosis could help address an existing knowledge gap in the field and reveal potential for future saproxylic invertebrate management.

## 2. Material and Methods

### 2.1. Insect Collection and the Isolation of Yeasts

**Figure 1:**
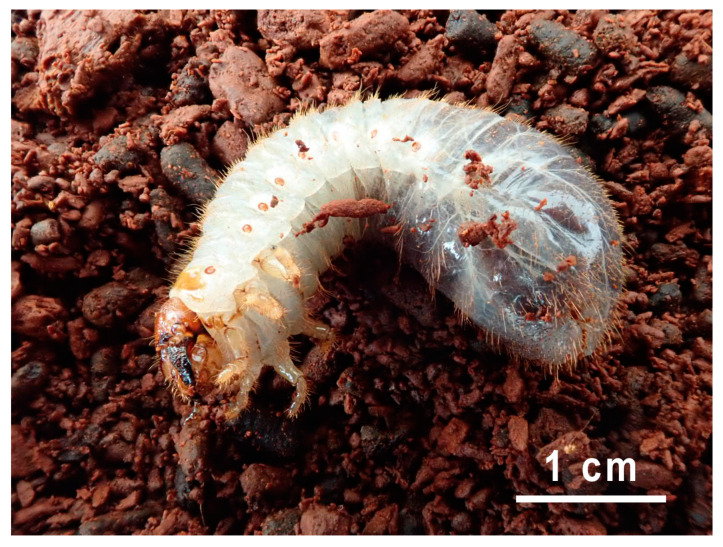
*Osmoderma barnabita* Larva 1 on rotten wood (photo S. A. Davenis).

Two hermit-like L3-praepupa instar larvae (Figure 1) were collected from a decaying oak log (Table 1).

The larvae were maintained in the same rotten wood at 10–15 °C until they were required for examination (Figure 1). The larvae were placed in Petri dishes for 1–3 days without food prior to dissection.

In order to isolate the yeasts, the larvae were placed in a −20 °C freezer for approximately 10 min and, after removal, they were surface sterilized with 70% ethanol for 1 min, washed twice in sterile phosphate-buffered saline (1× PBS, pH 7.4) to remove contaminates and the ethanol, and then dried for 1 min. The preparation of the intestinal tracts of the larvae was performed on a sterilized glass slide with a pair of sterile tweezers and a scalpel under sterile conditions. The whole gut was removed and washed twice with sterile 1× PBS. The gut was divided into three regions: the foregut, the midgut, and the hindgut. Each region was separated and homogenized using a small handheld plastic pestle in 1.5 mL tubes containing 200 µL sterile 1× PBS [34]. Immediately after homogenizing and shaking, a series of 10-fold dilutions of the suspension were performed, and appropriate dilutions were plated on YM agar with antibiotics (containing yeast extract, malt extract, peptone, and glucose) at pH 3.5 (YM agar: 3 g/L yeast extract, 3 g/L malt extract, 5 g/L glucose, 0.1 g/L chloramphenicol, and 25 g/L agar) [34,35]. Rotten wood samples were collected from larval galleries in both locations and were mixed in a 1:1 ratio. The mixed rotten wood sample (10 g) was suspended in 90 mL of sterile water in a 250 mL Erlenmeyer flask and shaken on a rotary shaker at 24 ± 1 °C for 1 h to detach the cells. The samples were serially diluted, and 100 µL of the diluted samples was spread onto YM agar. The plates were incubated at 25 °C for 5 days. Afterwards, the numbers of colony-forming units (CFU/mL) were determined. The yeasts were purified and maintained on Sabouraud dextrose agar (SDA; Liofilchem, Roseto degli Abruzzi (TE), Italy) slants at 4 °C [34].

### 2.2. Phenotypic Characterization of Yeast

The morphology, biochemistry, and physiology of the yeast isolates were characterized using standards commonly employed in yeast taxonomy studies [34]. For the purpose of micromorphological characterization and morphological studies, the cultures were grown on 2% MEA medium (malt extract agar: 20 g/L malt extract, 20 g/L glucose, 1 g/L peptone, 0.01 g/L ZnSO_4_ × 7H_2_O, 0.005 g/L CuSO_4_ × 5H_2_O, and 25 g/L agar) [36] and 4% glucose–peptone–yeast extract medium (GPY agar: 40 g/L glucose, 5 g/L peptone, 5 g/L yeast extract, and 20 g/L agar) at 25 °C for a period ranging from 3 to 7 days. After incubation, cell morphology was studied using a light microscope (Leica DM 5000 B, Leica, Wetzlar, Germany) equipped with a DFC 450 camera (Leica, Wetzlar, Germany).

### 2.3. DNA Extraction PCR and Sequencing

Prior to DNA extraction, the yeasts were cultured on SDA at 30 °C for 42 h. The yeast cells were harvested from agar cultures and resuspended in 200 µL of PBS. The DNA was extracted and purified using a ZR Fungal/Bacterial DNA MiniPrep kit and a DNA purification kit (Zymo Research, Tustin, CA, USA), following the instructions provided by the manufacturer. The isolates were identified by sequencing the large subunit (26S) rDNA (D1/D2 domain of the LSU). PCR amplification was conducted in a 25-µL reaction volume containing final concentrations of KAPATaq Ready Mix (KAPAbiosystems, Wilmington, DE, USA), 0.4 µM of each NL1 and NL4 [37] primer, and 1 µL of template DNA. The target region was amplified by PCR using a Techne TC-5000 Thermocycler (Bibby Scientific Ltd., Stone, UK). Amplification was performed with initial denaturation at 95 °C for 2 min, followed by 36 cycles of denaturation at 94 °C for 1 min, annealing at 52 °C for 30 s, extension at 72 °C for 2 min, and final extension at 72 °C for 7 min [38]. The PCR products were purified using exonuclease I (Thermo Scientific, Waltham, MA, USA) and Shrimp Alkaline Phosphatase (Thermo Scientific, Waltham, MA, USA), according to Stepień et al. (2019) [39]. The sequencing of purified PCR products was performed using BaseClear B.V. (Leiden, The Netherlands). The PCR products from both the 5′ and 3′ ends were sequenced for each sample using the same primer set as the initial amplification. The obtained sequences were assembled and edited using the program BioEdit version 7.0.5.3. For species identification, the sequences (~500 bp) were compared with publicly available sequences in the National Center for Biotechnology Information (NCBI; https://blast.ncbi.nlm.nih.gov/Blast.cgi, accessed on 26 March 2024) database using the BLAST algorithm. Two sequences were considered to belong to the same species if they showed at least 99% similarity.

The identified isolates were visualized using a heatmap and a Venn diagram (package version 1.2.2) using RStudio version 4.2.3 [40]. The heatmap was generated using the heatmap function of the heatmap package version 1.0.12 [41], whereas the Venn diagram was created using the ggVennDiagram function of the ggVennDiagram package version 1.2.2 [42].

### 2.4. Phylogenetic Analysis

Evolutionary analyses were conducted using MEGA 11 software [43]. A total of 56 nucleotide sequences from Saccharomycetales were analyzed, with 36 obtained from the present study and 20 retrieved from GenBank (http://www.ncbi.nlm.nih.gov/genbank, accessed 25 March 2024). *Schizosaccharomyces pombe* Lindner was used as the outgroup in this analysis. The final dataset comprised 528 nucleotide-length sequences. The sequences were aligned using the ClustalW algorithm with MEGA 11 software, and the evolutionary history was inferred through maximum likelihood, employing the Tamura–Nei model [44]. To account for potential variations in the evolutionary rates across sites, a discrete gamma distribution was selected.

The analysis of Trichosporonaceae involved a total of 19 nucleotide sequences, 8 of which were obtained from the current study and 11 were retrieved from GenBank (http://www.ncbi.nlm.nih.gov/genbank, accessed 26 March 2024)*. Dioszegia crocea* (Buhagiar) M. Takash., T. Deák, and Nakase were designated as the outgroup. The final dataset comprised 568 nucleotide-length sequences, which were aligned using the ClustalW algorithm. The evolutionary history was determined using the maximum likelihood method with the Kimura-2 parameter model [45], which is a widely accepted approach in the field. In order to account for potential variations in the speed of evolution among the sites, a discrete gamma distribution was also utilized.

## 3. Results

**Figure 2:**
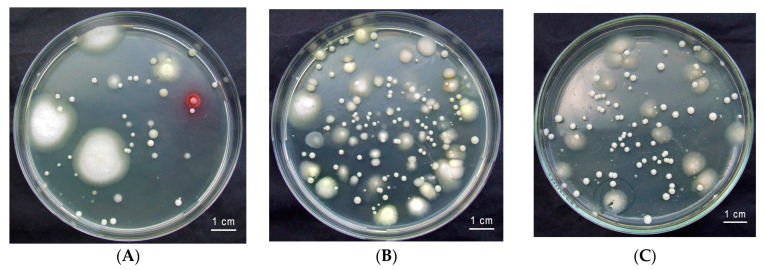
Morphological diversity of yeast from the gut of *O. barnabita* Larva 1: (**A**) foregut, (**B**) midgut, and (**C**) hindgut on YM agar.

Two *O. barnabita* larvae were collected from hollow oaks in Lithuania. The gut of *O. barnabita* larva consists of three compartments—the foregut (*stomodeum*), the midgut (*mesenteron*), and the hindgut (*proctodeum*). All gut compartments were colonized by yeast, with cell densities of (1.4 ± 0.1) × 10^4^ cells per ml in the midgut (Figure 2B), (12.5 ± 2.1) × 10^4^ cells per ml in the hindgut (Figure 2C), and (3.2 ± 0.2) × 10^1^ cells per ml in the foregut (Figure 2A), from larval galleries of (2.3 ± 0.3) × 10^1^ cells per mL.

The colonies on GPYA plates were white, tan, or cream colored, with glistening, smooth, or wrinkled surfaces, and appearing butyrous, friable, or mucoid, with entire or undulating margins.

**Figure 3:**
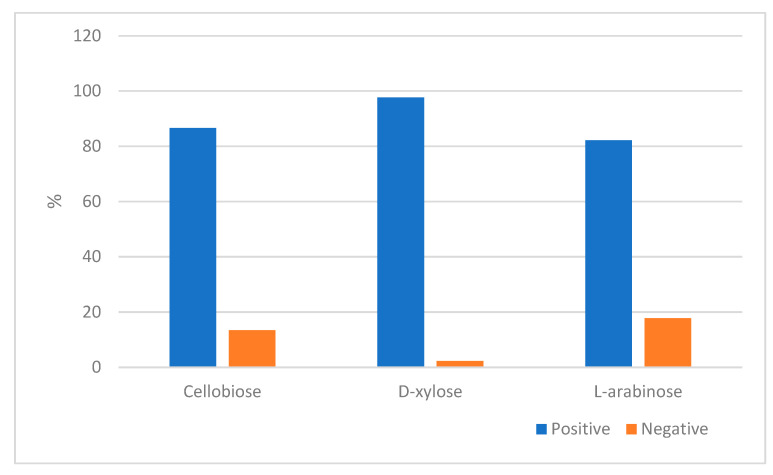
The ability of yeast to assimilate some carbon sources (in percentages).

The ability of the yeast species associated with *O. barnabita* larvae to utilize D-xylose, L-arabinose, and cellobiose aerobically, which are three carbon compounds and the main components of lignocellulose, are shown in Figure 3.

Almost all the yeasts were able to assimilate D-xylose (97.7%). Cellobiose and L-arabinose were utilized by 86.6% and 82.2% of the yeasts isolated from the gut of *O. barnabita*, respectively.

**Figure 4:**
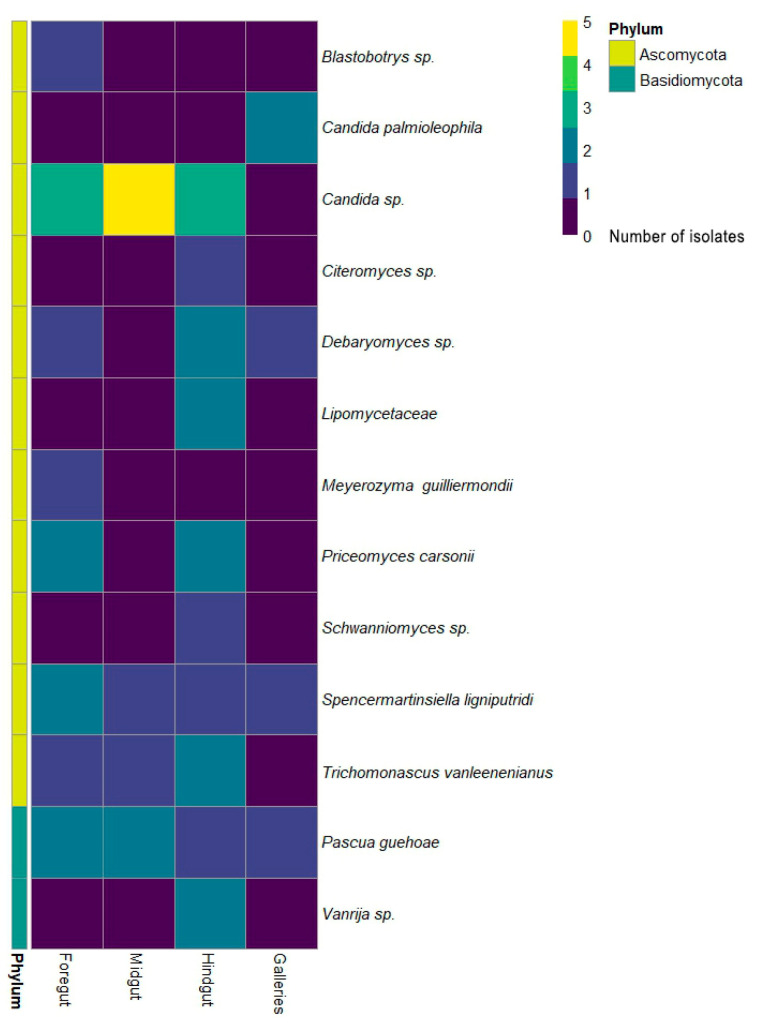
Heatmap of yeasts isolated from the gut of *Osmoderma barnabita* and the galleries.

The 45 yeast strains were obtained from the gut of both *O. barnabita* larvae, with 23 strains originating from Larva 1, 16 strains from Larva 2, and 6 strains from the galleries. Based on the sequence analysis of the rRNA gene D1/D2 region, as well as the morphology, biochemistry, and physiology, 37 strains were identified to be in Phylum *Ascomycota*, and 8 strains were identified to be in Phylum *Basidiomycota* (Figure 4). Species from the *Candida* genus were found in all gut compartments in both *O. barnabita* larvae.

**Figure 5:**
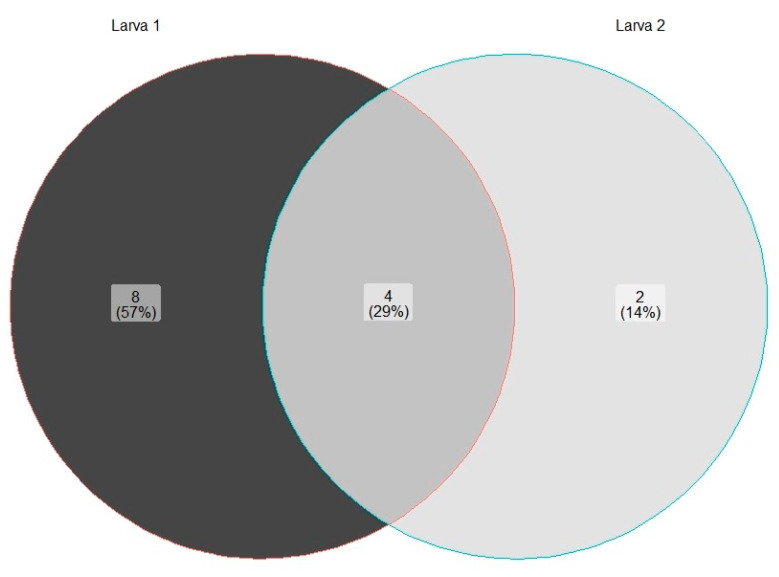
Percentage of yeast species isolated from each larva.

*Spencermartinsiella ligniputridi* and *Pascua guehoae* were detected in the foregut of both *O. barnabita* larvae and their galleries. *Debaryomyces* sp. was found only in the foregut and hindgut of Larva 1. *Spencermartinsiella ligniputridi*, *Pascua guehoae*, and *Candida* sp. were detected in the foregut of both *O. barnabita* larvae. *Trichomonascus vanleenenianus* was isolated from Larva 2 only. Two yeast isolates were assigned to the Lipomycetaceae family. These yeast isolates exhibited characteristics common to all genera of the Lipomycetaceae family, so more detailed studies are needed to determine the specific species. Figure 5 shows the number of yeast species isolated from each of the *O. barnabita* larva. Out of 16 yeast species, 8 (57%) were isolated from Larva 1 only. Yeast species from the *Candida*, *Spencermartinsiella*, and *Pascua* genera, as well as yeasts from the Lipomycetaceae family, were found in the gut of both *O. barnabita* larvae.

**Figure 6:**
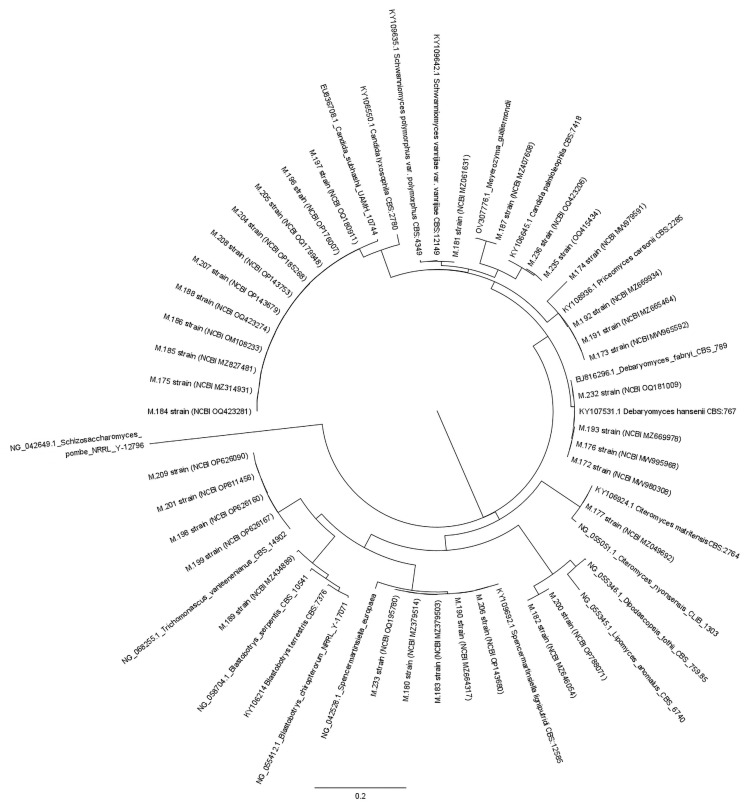
Phylogenetic relationships among ascomycetous yeast genera.

**Figure 7:**
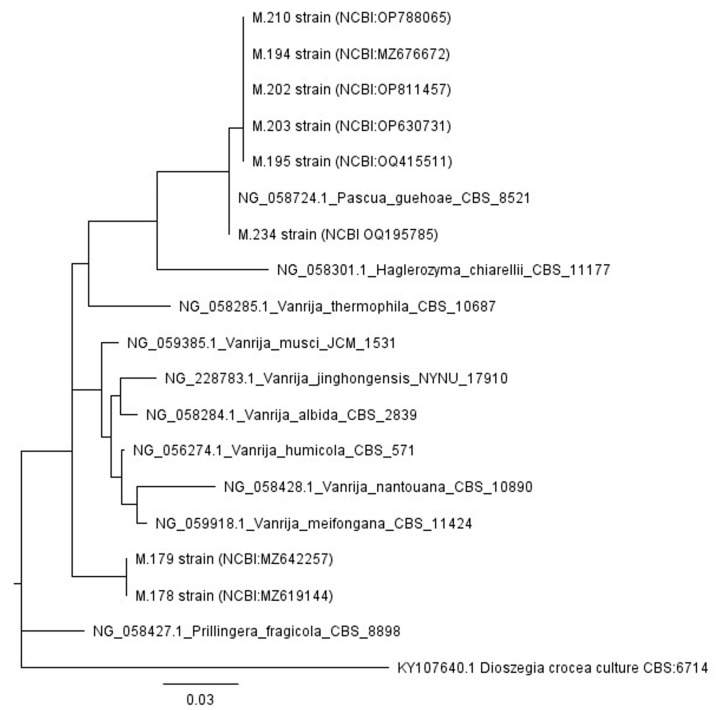
Phylogenetic relationships among basidiomycetous yeast genera.

The analysis of the phylogenetic relationships among ascomycetous (Figure 6) and basidiomycetous (Figure 7) yeast genera, using the large subunit (26S) rDNA (D1/D2 domain of the LSU), indicates several key findings. Figure 6 demonstrates that most strains were identified up to the genera or family level. Only a few strains were identified as belonging to a specific species, such as *Meyerozyma guilliermondii*, *Candida palmioleophila*, *Priceomyces carsonii*, *Spencermartinsiella lingiputridi*, or *Trichomonascus vanleenenianus* (Figure 6). The Phylogenetic relationships demonstrated in Figure 7 indicate that six strains were assigned to the *Pascua guehoae* species. Moreover, two strains (M.179 and M.178) were closely related to the *Vanrija* genus, but their precise species could not be identified.

## 4. Discussion

A veteran tree, as defined by Read (2000) [46], is one that captures the interest of the public due to its age, size, or condition. In addition to mere survival, it symbolizes resilience, having endured far beyond the typical lifespan expected of its species. Lonsdale (1999) [47] acknowledges the diverse lifespans of tree species and their various entry points into old age, recognizing them as pivotal elements supporting biodiversity within wooded landscapes. These venerable trees serve as sanctuaries for a plethora of life forms, due to their role in preserving entire ecosystems. Among them, the oak stands out as more than just a forest dweller. Oaks, with their remarkable longevity, can reach ages of up to 1000 years, as documented by Skarpaas et al. (2017) [48] and Sverdrup-Thygeson et al. (2017) [49]. Veteran trees form intricate habitats renowned for their rich and specialized biodiversity, housing a multitude of rare and endangered species. Among these inhabitants are hermit beetles, whose primary abodes are the broad-leaved old-growth forests abundant in hollow trees and decaying wood, as noted by Maurizi et al. (2017) [50]. Dead wood plays a major role in forest ecosystems as it stores carbon, nutrients, and water, influences soil development and regeneration, and serves as a reservoir of biodiversity by retaining complex trophic chains and providing microhabitats for a broad diversity of organisms, including saproxylic species [2,51]. Saproxylic arthropod communities are components of forest biodiversity that contribute to the decomposition of fallen trees and nutrient cycling [52].

The seemingly mundane activity of numerous saproxylophagous beetle larvae, which specialize in decomposing and consuming wood, results in the enlargement of their habitat through the process of feeding on wooden walls. This transformation occurs as the larvae consume the wood, which is then excreted as a mixture of frass, excrement, and remains. These materials gather at the cavity’s base, along with external contributions of leaves, branches, and seeds [16]. The trophic positions of saproxylic insects depend on the stage of wood decomposition, and it seems that microbial biomass is important in their diets. Microbial biomass can form a more important part of the diet of consumers than the dead plant material itself in food webs based on dead rather than living autotrophs [52].

The *Osmoderma eremita* species complex is known to thrive within the hollows of mature oaks, limes, beeches, and various other deciduous tree species, including fruit trees. They have been observed to inhabit both natural forests and urban environments, as noted by Siitonen (2012) [53], and have been found to be particularly responsive to forest management practices, as highlighted by Smolis et al. (2023) [54]. This beetle species is of significant importance in environmental research as it serves as an indicator of the richness of saproxylic beetle species within tree hollows. Referred to as an ‘umbrella species’ by Ranius (2002) [16], its presence often signifies the overall health and biodiversity of its habitat. Two *O. barnabita* larvae were collected from veteran oak trees in Lithuania with the objective of estimating the species composition of cultivable yeasts inhabiting their guts and identifying them. However, it should be noted that *O. barnabita* is included in the Bern Convention (Annex II), EU Habitat Directive (Annexes II and IV), and the Red Data Book of Lithuania as a vulnerable species [19,20], which represents a significant limitation to this study.

The digestive tract of insects comprises three primary regions: the foregut (*stomodeum*), the midgut (*mesenteron*), and the hindgut (*proctodeum*) [55,56,57]. These regions exhibit anatomical variations based on the insect group and their dietary habits. Some groups possess crypts, caeca, or enlargements that facilitate the retention of microorganisms within the tract [57,58]. For example, the larvae of *O. eremita* possess a fermentation chamber that houses nitrogen-fixing bacteria [59]. Apart from anatomical distinctions, these intestinal compartments serve different functions and have varying pH levels, creating diverse environments conducive to microbial colonization. However, the exact functions of these gut symbionts remain understudied [23].

Following meticulous dissection of the digestive tract, which was then diluted, we successfully obtained yeast symbiont colonies from each section of the gut of the *O. barnabita* larvae. As observed by Davis in 2015 [60], the presence of yeast extends to all stages of the beetle’s life cycle. The yeast symbionts are consistently identified in a range of tissues and organs, including the integuments and mycangial structures of adults, larvae, and pupae, the oviposition galleries, pupal chambers, digestive tracts, and vascular tissues of larvae and adults, and the phloem and xylem vascular tissues [58].

The literature on the *O. eremita* species complex predominantly describes it as saproxylophagous, meaning that it feeds primarily on dead wood (REF). However, recent evidence from the last decade suggests a shift in understanding. There is growing evidence that beetle larvae may be primarily polyphagous, with its primary food source being the loose organic debris that accumulates in tree cavities, as demonstrated by Landvik et al. (2016) [61]. The results of our study further support this notion.

Yeast colony forming unit (CFU/mL) levels were observed to be highest in the hindgut ((12.5 ± 2.1) × 10^4^ cells per mL) and lowest in the foregut ((3.2 ± 0.2) × 10^1^ cells per mL). For example, in the green lacewing *Chrysoperla rufilabris*, yeast abundance was higher in the diverticulum (3.7 × 10^3^ CFUs) and foregut (1.6 × 10^3^ CFUs) than in the midgut (2.0 × 10^2^ CFUs) and hindgut (8.3 × 10^1^ CFUs) [62]. In the hindguts of *Aegus subnitidus* female adults, yeasts were isolated, ranging from 6.7 × 10 to 4.1 × 10^4^ CFU/hindgut, and in instar larvae of *Aegus subnitidus*, ranging from 5.8 ×10^2^ to 1.4 × 10^4^ CFU/mL-galleries [63]. In healthy stingless bee (*Tetragonisca angustula*) adults, the amounts of yeasts ranged from 10^4^ CFU/mL to 10^6^ CFU/mL [58]. This is consistent with existing knowledge that wood feeders and detractors tend to have the highest ratios of total gut microbial biomass, often due to compartmentalized guts or enlarged hindguts, as highlighted by Engel and Moran (2013) [57]. Various insects, such as termites [64], detritus-feeding fly larvae [65], and scarab beetle larvae [66,67], all possess a dilated hindgut region that forms an anoxic fermentation chamber. In several species, including soil-dwelling scarab beetle larvae (Melolonthinae and Cetoniinae), the hindgut microbial community has been found to be highly diverse, and numerous gut microorganisms are consistently present, suggesting a level of symbiosis [66,67,68]. In contrast, the midgut of insects hosts a dense and diverse microbial community. This organ is the primary site of digestion and absorption in insects [62].

Insects contain a broad variety of microorganisms in their digestive tracts. According to the literature, many bacterial phyla, such as *Proteobacteria*, *Firmicutes*, *Bacteroidetes*, and others, are often found in the guts of insects [69,70,71,72,73]. Fungi from *Ascomycota* and *Basidiomycota* are the predominant in insect guts [69,72]. Yeast species, including *Candida*, *Debaryomyces*, Metschnikowia, and *Pichia*, were widely found across all developmental stages of insects [58,74]. We found *Candida* and *Debaryomyces* genera in the gut of *O. barnabita* larvae.

*Saccharomycetales* and *Trichosporonales* emerged as the predominant orders of cultivable yeasts found in the gut of *O. barnabita* larvae, according to the methodology used in this study. In this study, members of *Ascomycota* (82.2%) were predominant in the gut of *O. barnabita* larvae. In the guts of larvae such as *Oryctes nasicornis*, *Amphimallon solstitiale*, and *Hermetia illucens*, most of the obtained fungal OTUs were related to the phyla Ascomycota (17–99% of the total reads), *Basidiomycota* (0–47%), and *Zygomycota* (0–68%) [70,71]. In the gut of *Pelidnota luridipes* larvae, yeasts contributed to only 2.3% of the average abundance of cultivable microorganisms and are represented by species of the orders *Saccharomycetales* (99.74% of CFUs/mg of yeasts) and *Trichosporonales* (0.26% of CFUs/mg of yeasts) [70].

Insect-associated yeast communities are mainly composed of *Ascomycota* and *Saccharomycotina* [62]. According to our identification results of 45 yeast strains, it was found that most of the species were related to *Ascomycota*, with most of them belonging to the order *Saccharomycetales*. Yeasts of the genera of *Candida*, *Debaryomyces, Meyerozyma*, *Priceomyces*, *Schwanniomyces, Spencermartinsiella, Trichomonascus*, and *Blastobotrys* were present in the gut of *O. barnabita* larvae. Species of the *Trichosporonales* order represented the *Basidiomycota* phylum. *Candida* sp. stand out as one of the most abundant yeasts in the gut of both *O. barnabita* larvae and galleries. The *Candida* genus is highly polyphyletic, containing approximately 300 species distributed across more than 30 phylogenetic clades, which are linked to several presently accepted genera and 17 unaffiliated clades [75]. *Candida* species have been frequently isolated from the gut of adult insects [24,58,74,76], as well as from the gut of wood-boring larvae [77]. *Candida* species are predominant in the gut of *Dendroctonus armandi* at different developmental stages and in its galleries [78], as well as in *Pelidnota luridipes* larvae [70] and in the larvae of *Bactrocera dorsalis* [74]. Also, *Candida* strains are widely distributed across different environments, including plant material, soil, fresh and sea water, fungi, and the atmosphere [79]. Certain *Candida* species are closely related to saproxylic insects and have the ability to convert D-xylose and other key components of lignocellulose into ethanol [80,81]. *S. ligniputridi* and *P. guehoae* were detected in the foregut of both *O. barnabita* larvae and their galleries. *S. ligniputridi* has been found in rotten wood [82] and *P. guehoae* has been isolated from dung beetles [83].

To conclude, we isolated and identified common cultivable yeast associates of *O. barnabita* larvae for the first time. Also, our characterization of the yeast communities here was based solely on culture-dependent methods. Studies have shown that these techniques are often not representative of true communities due to biases in their growth on prepared media and the often biotrophic nature of arthropod-associated microbes (e.g., Rani et al. 2009) [84]. Although not without biases, culture-independent methods should be used in future studies to elucidate the full complement of fungi and other microbes involved in this multi-taxon symbiosis.

## 5. Conclusions

Two hermit-like L3-praepupa instar larvae were collected from a decaying oak log in Lithuania. The isolation, morphology, biochemistry, and physiology of the yeast isolates were characterized using standards commonly employed in yeast taxonomy studies. The isolates were identified by sequencing the large subunit (26S) rDNA (the D1/D2 domain of the LSU). All the gut compartments were colonized by yeast. The 45 yeast strains were obtained from the gut of both *O. barnabita* larvae, with 23 strains originating from Larva 1, 16 strains from Larva 2, and 6 strains from the galleries. According to our identification results of 45 yeast strains, it was found that most of the species were related to *Ascomycota*, with most of them belonging to the order Saccharomycetales. Yeasts of the genera of *Candida*, *Debaryomyces*, *Meyerozyma*, *Priceomyces, Schwanniomyces, Spencermartinsiella*, *Trichomonascus*, and *Blastobotrys* were present in the gut of *O. barnabita* larvae. From the *Basidiomycota* phylum, yeasts from the genera *Pascua* and *Vanrija* were found. Research on *Osmoderma* species could contribute to our understanding of the evolutionary mechanisms associated with the beetle–fungus–tree system. This is a fascinating biological system that can shed light on the community ecology and biogeography of yeasts.

## Figures and Tables

**Table 1:** Details of the *O. barnabita* larvae used in this study.

Sample	Site	Coordinates WGS84		Host	Collection Date	Body Weight, g	Body Length, cm	Collector
		Longitude	Latitude					
Larva 1	Daudžgiriai Manor Park, Biržai district	56.168662	24.650705	*Quercus robur*	10 July 2018	9.049	5.7	Povilas Ivinskis
Larva 2	Kaunas (Vytautas) Oak Park, Kaunas	54.896247	23.931999	*Quercus robur*	31 July 2019	7.844	5.5	Sigitas Algis Davenis

## Data Availability

The raw data supporting the conclusions of this article will be made available by the authors on request.

## References

[1] Tláskal V., Zrustová P., Vrška T., Baldrian P. (2017). Bacteria associated with decomposing dead wood in a natural temperate forest. FEMS Microbiol. Ecol..

[2] Speight M.C.D. (1989). Saproxylic invertebrates and their conservation. Nat. Environ. Ser..

[3] Fowles A.P., Alexander K.N.A., Key R.S. (1999). The Saproxylic Quality Index: Evaluating wooded habitats for the conservation of dead-wood Coleoptera. Coleopterist.

[4] Cavalli R., Mason F. (2003). Techniques for Re-Establishment of Dead Wood for Saproxylic Fauna Conservation.

[5] Speight M.C.D., Good J.A., Mason F., Nardi G., Tisato M. (2003). Development of eco-friendly forestry practices in Europe and the maintenance of saproxylic biodiversity. Proceedings of the International Symposium “Dead Wood: A Key to Biodiversity”, Mantova, Italy, 29–31 May 2003.

[6] Alexander K.N.A. (2008). Tree biology and saproxylic Coleoptera: Issues of definitions and conservation language. Rev. d’Ecologie.

[7] Dodelin B., Gaudet S., Fantino G. (2017). Spatial analysis of the habitat and distribution of *Osmoderma eremita* (Scop.) in trees outside of woodlands. Nat. Conserv..

[8] Ratcliffe B. (1977). Descriptions of the Larva and Pupa of *Osmoderma subplanata* (Casey) and *Cremastocheilus wheeleri* LeConte (Coleoptera: Scarabaeidae). J. Kans. Entomol. Soc..

[9] Baraud J., Tauzin P. (1991). Une nouvelle espèce Europeenne du genre *Osmoderma* Serville. Lambillionea.

[10] Sparacio I. (2000). *Osmoderma europee* con descrizione di una specie dell’ italia meridionale. Nat. Sicil..

[11] Stegner J.W. (2002). Der Eremit, *Osmoderma eremita* (Scopoli, 1763) (Coleoptera: Scarabaeidae), in Sachsen: Anforderungen an Schutzmaßnahmen für eine prioritäre Art der FFH-Richtlinie. [The Hermit-beetle *Osmoderma eremita* (Scopoli, 1763) (Coleoptera: Scarabaeidae) in Saxony: Required protective measures for a priority species in the sense of the habitats-directive]. Entomol. Nachrichten Berichte.

[12] Bayartogtokh B., Kim J.I., Bae Y.J. (2012). Lamellicorn beetles (Coleoptera: Scarabaeoidea) in Korea and Mongolia. Entomol. Res..

[13] Webster R.P., Sweeney J.D., DeMerchant I. (2012). New Coleoptera records from New Brunswick, Canada: Geotrupidae and Scarabaeidae. ZooKeys.

[14] Bezborodov V.G. (2015). The genus *Osmoderma* (Coleoptera, Scarabaeidae, Trichiinae) in Siberia and the Russian Far East. Entomol. Rev..

[15] Bezborodov V.G. (2018). Lamellicorn Beetles (Coleoptera, Scarabaeoidea) of Manchuria (China): Fauna, Ecology, and Zoogeography. Entomol. Rev..

[16] Ranius T. (2002). *Osmoderma eremita* as an indicator of species richness of beetles in tree hollows. Biodivers. Conserv..

[17] Audisio P., Brustel H., Carpaneto G.M., Coletti G., Mancini E., Piattella E., Trizzino M., Dutto M., Antonini G., De Biase A. (2007). Updating the taxonomy and distribution of the European *Osmoderma*, and strategies for their conservation (Coleoptera, Scarabaeidae, Cetoniinae). Fragm. Entomol..

[18] Alexander K.N.A., Buche B., Dodelin B., Schlaghamersky J. *Osmoderma barnabita*. The IUCN Red List of Threatened Species 2010: e.T157901A5169119.

[19] Rašomavičius V. (2021). Red Data Book of Lithuania. Animals, Plants, Fungi.

[20] Ivinskis P., Davenis S.A., Rimšaitė J. Status and distribution of *Osmoderma barnabita* Motschulsky, 1845 (Coleoptera) in Lithuania. Proceedings of the 2nd International Conference “Smart Bio“.

[21] Hulcr J., Barnes I., De Beer Z.W., Duong T.A., Gazis R., Johnson A.J., Jusino M.A., Kasson M.T., Li Y., Lynch S. (2020). Bark beetle mycobiome: Collaboratively defined research priorities on a widespread insect-fungus symbiosis. Symbiosis.

[22] Blackwell M., Heitman J., Howlett B.J., Crous P.W., Stukenbrock E.H., Timothy Y., James T.Y., Gow N.A.R. (2017). Made for each other: Ascomycete yeasts and insects. The Fungal Kingdom.

[23] Birkemoe T., Jacobsen R.M., Sverdrup-Thygeson A., Biedermann P.H.W., Ulyshen M.D. (2018). Insect-Fungus Interactions in Dead Wood Systems. Saproxylic Insects Diversity, Ecology and Conservation.

[24] Chakraborty A., Modlinger R., Ashraf M.Z., Synek J., Schlyter F., Roy A. (2020). Core Mycobiome and Their Ecological Relevance in the Gut of Five Ips Bark Beetles (Coleoptera: Curculionidae: Scolytinae). Front. Microbiol..

[25] Wang Z., Liu Y., Wang H., Meng X., Liu X., Zhang X., Lu Q. (2020). Correction to: Ophiostomatoid fungi associated with *Ips subelongatus*, including eight new species from northeastern China. IMA Fungus.

[26] Gadanho M., Libkind D., Sampaio J.P. (2006). Yeast diversity in the extreme acidic environments of the Iberian pyrite belt. Microb. Ecol..

[27] Burgaud G., Arzur D., Durand L., Cambon-Bonavita M.A., Barbier G. (2010). Marine culturable yeasts in deep-sea hydrothermal vents: Species richness and association with fauna. FEMS Microbiol. Ecol..

[28] Cantrell S.A., Dianese J.C., Fell J., Gunde-Cimerman N., Zalar P. (2011). Unusual fungal niches. Mycologia.

[29] Buzzini P., Branda E., Goretti M., Turchetti B. (2012). Psychrophilic yeasts from worldwide glacial habitats: Diversity, adaptation strategies and biotechnological potential. FEMS Microbiol. Ecol..

[30] Chung D., Kim H., Choi H.S. (2019). Fungi in salterns. J. Microbiol..

[31] Nagano Y., Miura T., Tsubouchi T., Lima A.O., Kawato M., Fujiwara Y., Fujikura K. (2020). Cryptic fungal diversity revealed in deep-sea sediments associated with whale-fall chemosynthetic ecosystems. Mycology.

[32] Cadete R.M., Lopes M.R., Rosa C.A., Buzzini P., Lachance M.A., Yurkov A. (2017). Yeasts Associated with Decomposing Plant Material and Rotting Wood. Yeasts in Natural Ecosystems: Diversity.

[33] Suh S., Blackwell M. (2005). The beetle gut as a habitat for new species of yeasts. Insect–Fungal Associations. Ecol. Evol..

[34] Suh S.O., Marshall C.J., McHugh J.V., Blackwell M. (2003). Wood ingestion by passalid beetles in the presence of xylose-fermenting gut yeasts. Mol. Ecol..

[35] Kurtzman C.P., Fell J.W., Boekhout T. (2011). The Yeasts, a Taxonomic Study.

[36] Frisvad J.C. (2012). Media and growth conditions for induction of secondary metabolite production. Methods Mol. Biol..

[37] Leaw S.N., Chang H.C., Sun H.F., Barton R., Bouchara J.P., Chang T.C. (2006). Identification of medically important yeast species by sequence analysis of the internal transcribed spacer regions. J. Clin. Microbiol..

[38] Boekhout T., Robert V. (2002). Yeast in Food Beneficial and Detrimental Aspects.

[39] Stepień Ł., Gromeradzka K., Chełkowski J., Basińska-Barczak A., Lalak-Kończugowska J. (2019). Diversity and mycotoxin production by *Fusarium temperatum* and *Fusarium subglutinans* as casual agents of pre-harvest *Fusarium* maize ear rot in Poland. J. Appl. Genet..

[40] RStudio Team (2020). RStudio: Integrated Development for R.

[41] Kolde R. (2013). A Package for Drawing Pretty Heatmaps in R. Pheatmap: Pretty Heatmaps. https://github.com/raivokolde/pheatmap.

[42] Gao C.-H., Yu G., Cai P. (2021). ggVennDiagram: An Intuitive, Easy-to-Use, and Highly Customizable R Package to Generate Venn Diagram. Front. Genet..

[43] Tamura K., Stecher G., Kumar S. (2021). MEGA11: Molecular Evolutionary Genetics Analysis Version 11. Mol. Biol. Evol..

[44] Tamura K., Nei M. (1993). Estimation of the number of nucleotide substitutions in the control region of mitochondrial DNA in humans and chimpanzees. Mol. Biol. Evol..

[45] Kimura M. (1980). A simple method for estimating evolutionary rates of base substitutions through comparative studies of nucleotide sequences. J. Mol. Evol..

[46] Read H. (2000). Veteran Trees: A Guide to Good Management.

[47] Lonsdale D. (1999). Principles of Tree Hazard Assessment and Management. Research for Amenity Trees No. 7.

[48] Skarpaas O., Blumentrath S., Evju M., Sverdrup-Thygeson A. (2017). Prediction of biodiversity hotspots in the Anthropocene: The case of veteran oaks. Ecol. Evol..

[49] Sverdrup-Thygeson A., Skarpaas O., Blumentrath S., Birkemoe T., Evju M. (2017). Habitat connectivity affects specialist species richness more than generalists in veteran trees. For. Ecol. Manag..

[50] Maurizi E., Campanaro A., Chiari S., Maura M., Mosconi F., Sabatelli S., Zauli A., Audisio P., Carpaneto G.M. (2017). Guidelines for the monitoring of *Osmoderma eremita* and closely related species. Nat. Conserv..

[51] Tsikas A., Karanikola P. (2022). To Conserve or to Control? Endangered Saproxylic Beetles Considered as Forest Pests. Forests.

[52] Garrick R.C., Reppel D.K., Morgan J.T., Burgess S., Hyseni C., Worthington R.J., Ulyshen M.D. (2019). Trophic interactions among dead-wood-dependent forest arthropods in the southern Appalachian Mountains, USA. Food Webs.

[53] Siitonen J., Stokland J., Siitonen J., Jonsson B.G. (2012). Microhabitats. Biodiversity in Dead Wood.

[54] Smolis A., Zając K., Tyszecka K., Kadej M. (2023). Why is the hermit beetle so rare in Central European managed forests? Habitat requirements of the forest population of *Osmoderma barnabita*. For. Ecol. Manag..

[55] Ross H.H., Ross C.A., Ross J.R.P. (1982). A Textbook of Entomology.

[56] Lopez-Guerrero I. (2002). Anatomy and Histology of the Digestive System of *Cephalodesmius armiger* Westwood (Coleoptera, Scarabaeidae, Scarabaeinae). Coleopt. Bull..

[57] Engel P., Moran N.A. (2013). The gut microbiota of insects—Diversity in structure and function. FEMS Microbiol. Rev..

[58] Stefanini I. (2018). Yeast-insect associations: It takes guts. Yeast.

[59] Jönsson N., Méndez M., Ranius T. (2004). Nutrient richness of wood mould in tree hollows with the Scarabaeid beetle *Osmoderma eremita*. Anim. Biodivers. Conserv..

[60] Davis T.S. (2015). The Ecology of Yeasts in the Bark Beetle Holobiont: A Century of Research Revisited. Microb. Ecol..

[61] Landvik M., Niemelä P., Roslin T. (2016). Mother knows the best: An essential role for non-wood dietary components in the life cycle of a saproxylic scarab beetle. Oecologia.

[62] Malassigné S., Minard G., Vallon L., Martin E., Moro C.V., Luis P. (2021). Diversity and Functions of Yeast Communities Associated with Insects. Microorganisms.

[63] Yamamoto D., Toki W. (2023). Presence of non-symbiotic yeasts in a symbiont-transferring organ of a stag beetle that lacks yeast symbionts found in other stag beetles. Sci. Rep..

[64] Hongoh Y. (2011). Toward the functional analysis of uncultivable, symbiotic microorganisms in the termite gut. Cell. Mol. Life Sci..

[65] Cook D.M., DeCrescenzo Henriksen E., Upchurch R., Peterson J.B.D. (2007). Isolation of polymer-degrading bacteria and characterization of the hindgut bacterial community from the detritus-feeding larvae of *Tipula abdominalis* (Diptera: Tipulidae). Appl. Environ. Microbiol..

[66] Andert J., Marten A., Brandl R., Brune A. (2010). Inter- and intraspecific comparison of the bacterial assemblages in the hindgut of humivorous scarab beetle larvae (*Pachnoda* spp.). FEMS Microbiol. Ecol..

[67] Huang S., Zhang H. (2013). The impact of environmental heterogeneity and life stage on the hindgut microbiota of *Holotrichia parallela* larvae (Coleoptera: Scarabaeidae). PLoS ONE.

[68] Egert M., Stingl U., Bruun L.D., Pommerenke B., Brune A., Friedrich M.W. (2005). Structure and topology of microbial communities in the major gut compartments of *Melolontha melolontha* larvae (Coleoptera: Scarabaeidae). Appl. Environ. Microbiol..

[69] Ravenscraft A., Berry M., Hammer T., Peay K., Boggs C. (2019). Structure and function of the bacterial and fungal gut microbiota of Neotropical butterflies. Ecol. Monogr..

[70] Falqueto S.A., Rosa de Sousa J., Correla da Silva R., Ferreira da Silva G., Daniel Guariz Pinheiro D.G., Soares M.A. (2022). Larval gut microbiome of *Pelidnota luridipes* (Coleoptera: Scarabaeidae): High bacterial diversity, different metabolic profiles on gut chambers and species with probiotic potential. World J. Microbiol. Biotechnol..

[71] Klüber P., Müller S., Schmidt J., Zorn H., Rühl M. (2022). Isolation of Bacterial and Fungal Microbiota Associated with *Hermetia illucens* Larvae Reveals Novel Insights into Entomopathogenicity. Microorganisms.

[72] Zhang Z., Jiao S., Li X., Li M. (2018). Bacterial and fungal gut communities of *Agrilus mali* at diferent developmental stages and fed diferent diets. Sci. Rep..

[73] Mondal S., Somani J., Roy S., Babu A., Pandey A.K. (2023). Insect Microbial Symbionts:Ecology, Interactions, and Biological Significance. Microorganisms.

[74] Guo Q., Yao Z., Cai Z., Bai S., Zhang H. (2022). Gut fungal community and its probiotic effect on *Bactrocera dorsalis*. Insects Sci..

[75] Boekhout T., Amend A.S., Baidouri F.E., Gabaldón T., Geml J., Mittelbach M., Robert V., Tan C.S., Turchetti B., Vu D. (2022). Trends in yeast diversity discovery. Fungal Divers..

[76] Jacobsen R.M., Kauserud H., Sverdrup-Thygeson A., Bjorbækmo M.M., Birkemoe T. (2017). Wood-inhabiting insects can function as targeted vectors for decomposer fungi. Fungal Ecol..

[77] Hui F.L., Niu Q.H., Ke T., Liu Z. (2012). *Candida ficus* sp. nov., a novel yeast species from the gut of Apriona germari larvae. Int. J. Syst. Evol. Microbiol..

[78] Hu X., Li M., Chen H. (2015). Community structure of gut fungi during different developmental stages of the Chinese white pine beetle (*Dendroctonus armandi*). Sci. Rep..

[79] de Melo Pereira G.V., Maske B.L., de Carvalho Neto D.P., Karp S.G., De Dea Lindner J., Martin J.G.P., de Oliveira Hosken B., Soccol C.R. (2022). What Is *Candida* Doing in My Food? A Review and Safety Alert on Its Use as Starter Cultures in Fermented Foods. Microorganisms.

[80] Suh S.O., Nguyenn N.H., Blackwell M. (2005). Nine new *Candida* species near *C. membranifaciens* isolated from insects. Mycol. Res..

[81] Wang S.A., Li F.L., Bai F.Y. (2010). *Candida laoshanensis* sp. nov. and *Candida qingdaonensis* sp. nov., anamorphic, ascomycetous yeast species isolated from decayed wood. Int. J. Syst. Evol. Microbiol..

[82] Dlauchy D., Lee C.F., Péter G. (2012). *Spencermartinsiella ligniputridi* sp. nov., a yeast species isolated from rotten wood. Int. J. Syst. Evol. Microbiol..

[83] Nwaefuna A.E., Boekhout T., Aloy M.G., Vrhovsek U., Zhou N. (2023). Diversity of dung beetle-associated yeasts from pristine environments of Botswana. Yeast.

[84] Rani A., Sharma A., Rajagopal R., Adak T., Bhatnagar R.K. (2009). Bacterial diversity analysis of larvae and adult midgut microflora using culture-dependent and culture-independent methods in lab-reared and field-collected *Anopheles stephensi*-an Asian malarial vector. BMC Microbiol..

